# The DARS (Dopamine Augmented Rehabilitation in Stroke) trial: protocol for a randomised controlled trial of Co-careldopa treatment in addition to routine NHS occupational and physical therapy after stroke

**DOI:** 10.1186/1745-6215-15-316

**Published:** 2014-08-08

**Authors:** Bipin B Bhakta, Suzanne Hartley, Ivana Holloway, J Alastair Couzens, Gary A Ford, David Meads, Catherine M Sackley, Marion F Walker, Sharon P Ruddock, Amanda J Farrin

**Affiliations:** University of Leeds, Leeds, UK; NHS Grampian, Aberdeen, UK; Oxford University Hospitals NHS Trust, Oxford, UK; University of Oxford, Oxford, UK; University of East Anglia, Norwich, Norfolk, UK; University of Nottingham, Nottingham, UK

**Keywords:** Stroke, Rehabilitation, L-dopa, Mobility, Recovery, Double-blind, Placebo, Trial

## Abstract

**Background:**

Stroke has a huge impact, leaving more than a third of affected people with lasting disability and rehabilitation remains a cornerstone treatment in the National Health Service (NHS). Recovery of mobility and arm function post-stroke occurs through re-learning to use the affected body parts and/or learning to compensate with the lesser affected side. Promising evidence suggests that the addition of Co-careldopa to physical therapy and occupational therapy may improve the recovery of arm and leg movement and lead to improved function.

**Methods/design:**

Dopamine Augmented Rehabilitation in Stroke (DARS) is a multi-centre double-blind, randomised, placebo, controlled clinical trial of Co-careldopa in addition to routine NHS occupational therapy and physical therapy as part of early stroke rehabilitation. Participants will be randomised on a 1:1 basis to either Co-careldopa or placebo. The primary objective of the trial is to determine whether the addition of six weeks of Co-careldopa treatment to rehabilitation therapy can improve the proportion of patients who can walk independently eight weeks post-randomisation.

**Discussion:**

The DARS trial will provide evidence as to whether Co-careldopa, in addition to routine NHS occupational and physical therapy, leads to a greater recovery of motor function, a reduction in carer dependency and advance rehabilitation treatments for people with stroke.

**Trial registration:**

ISRCTN99643613 assigned on 4 December 2009.

## Background

Stroke is the most common cause of severe disability (annual UK incidence of first stroke is 100,000). Stroke has a huge impact, leaving more than a third of affected people with lasting disability affecting selfcare. One year after a stroke 31% of survivors are still dependent for outside mobility and 15% are dependent for inside mobility. The number of disabled stroke survivors will increase due to ageing population demographics. The cost of stroke accounts for 6% of the total National Health Service (NHS) and social services expenditures. Although acute stroke interventions, such as thrombolysis, can reduce mortality and morbidity, rehabilitation remains the cornerstone treatment within the NHS for the majority of people with stroke.

The role of high quality rehabilitation within comprehensive stroke services is widely acknowledged as described by the English National Stroke Strategy. Despite the clear benefits of organised stroke care at least 30,000 people in the UK each year are left with physical disability, increasing the long term societal costs of dependency and resulting in a major impact on the quality of life of those individuals and their families. Despite the clear benefits of organised stroke care, a third of people with stroke are left with significant physical disabilities. Physical and occupational therapies have been shown to benefit people but residual disability for a large proportion of patients still remains a key issue in regaining full independence.

Learning is an essential process by which recovery of mobility and arm function occurs after stroke, either through relearning to use the affected body parts and/or learning to compensate with the lesser affected side (for example, one-handed dressing). These situations involve the patient becoming attuned to the perceptions which guide skilled movement, such as vision and proprioception. At a clinical level, the patient practises motor skills with guidance and support from therapists [1]. At a biological level, this practice leads to changes in behaviour (learning) through functional re-organization of the central nervous system (CNS) by a process of neural plasticity [2].

Pharmacological priming of the brain and motor skill acquisition: The emerging evidence from pilot studies indicates that the addition of certain drugs with physical and occupational therapy may improve the recovery of arm and leg movements and, thus, recovery of essential day to day activities such as walking and getting dressed. These improvements are in addition to the benefits gained from physiotherapy and occupational therapy alone. These studies suggest that the nerve circuits in the brain respond better to the usual therapy when they are also exposed to drugs such as dopamine at the same time as having occupational or physiotherapy.

Evidence from animal and human studies indicates an important role of noradrenergic/dopaminergic brain pathways in motor skill acquisition [3, 4]. Animal studies demonstrate that neural plasticity comprises cellular processes (for example, changes in synaptic morphology, synaptic potentiation/depression, dendrite sprouting and alteration of axonal trajectories [5]). Involvement of adrenergic neurotransmitters in these processes raises the possibility of pharmacologically promoting neural plasticity by increasing catecholamine levels in the CNS (for example, oral amphetamine increases the brain levels of dopamine, serotonin and norepinephrine) which can modulate long-term changes in synaptic function. Studies in rats suggest that amphetamines can promote relearning after experimental brain injury [6]. Encouraged by this evidence, several clinical trials of amphetamines in stroke patients have been undertaken. A Cochrane review of 12 small clinical trials (344 patients) reported a trend towards improved motor function [7] and suggested that further studies to confirm an effect on motor recovery were warranted. There is no evidence of increased mortality/dependency with amphetamine administration in stroke patients. However, adverse sympathomimetic effects with amphetamines, such as tachycardia and hypertension, are reported.

A growing body of evidence suggests that learning and motor skill acquisition occurs through the dopaminergic system rather than through direct noradrenergic action of general arousal. Therefore, drugs that promote dopaminergic activity directly may be more appropriate as targeted brain modulators [8] in the context of motor skill acquisition and be associated with fewer adverse cardiovascular effects. Levodopa (L-dopa) is a precursor of dopamine which crosses the blood–brain barrier and is converted to dopamine in the brain. Co-careldopa is a routinely available inexpensive medication that will be used to deliver 100 mg of levodopa through its combination with 25 mg carbidopa. Co-careldopa is used to deliver L-dopa as this contains carbidopa, a peripheral dopa-decarboxylase inhibitor which reduces the peripheral adverse effects of levodopa. The peak effect is 0.5 to 2 hours after an oral dose and plasma half-life is 1 to 3 hours.

The impact of L-dopa on motor function in stroke has been investigated in small scale clinical studies taking into account the temporal linkage between drug administration and physical therapy treatment [9]. This randomized controlled trial (RCT) reported the effect of L-dopa (oral co-careldopa – 100 mg Levodopa/25 mg carbidopa) on motor function in 53 people who were three weeks to six months post stroke. All patients received daily physiotherapy sessions lasting 30 minutes for three weeks in a hospital setting. Motor function was assessed using the Rivermead Mobility Index (RMI). Significantly greater improvement in RMI scores and walking ability were reported in the L-dopa treated group compared with placebo. The drug was well tolerated and no serious drug related adverse events were reported. The effect on function was still present three weeks after cessation of L-dopa. Although these results are encouraging, the study has a number of limitations [10] including sample size and the recruitment of some patients in the post acute phase of stroke when effects on neuroplasticity may be less.

Rationale for the clinical trial: In this trial we will find out if the addition of Co-careldopa (a widely available and inexpensive form of the drug that is commonly used to treat Parkinson’s Disease) to routine NHS occupational and physical therapy enhance*s* the effect of the therapy and further improves recovery of functionally useful arm and leg movement in people with new or recurrent clinically diagnosed stroke. The dose and timing of the medication within this trial reflects current evidence on the use of L-dopa in this context [9, 11–13]. All study participants will receive usual stroke care within their hospital and community rehabilitation settings. The study drug will be used in addition to conventional rehabilitation treatment, including at home if rehabilitation treatment continues after discharge from hospital, up to a maximum of six weeks and no more than twice per day. Those potentially suitable to take part in this study will be identified on admission to hospital with stroke, and eligibility will be confirmed between day 5 and day 42 post-stroke.

## Methods/design

### Objectives

The primary objective relates to physical functioning and will compare the proportion of patients between treatment groups who are walking independently at eight weeks post-randomisation (as measured by a score of 7 or higher and who also answer ‘yes’ to item number 7 on the RMI).

Secondary objectives are to determine: (1) the impact on physical functioning and mood at eight weeks, six months and twelve months; (2) potential moderators and mediators of effect at eight weeks, six months and twelve months; (3) implementation within routine healthcare services; and (4) the cost-effectiveness of co-careldopa augmented rehabilitation for stroke compared to usual care within stroke services.

1. Investigation of impact on physical functioning and mood at eight weeks, six months and twelve months
  To compare the proportion of patients who are walking at six and twelve months post-randomisation between the groups (as measured by a score of 7 or higher on the RMI and who also answer yes on item number 7)
  To compare activities of daily living, mobility and dependency using the RMI (continuous), Barthel Index, Modified Rankin Scale, Nottingham Extended Activities of Daily Living (NEADL) scale and ABILHAND between groups.
  To compare psychological distress/mood between groups using the General Health Questionnaire 12 (GHQ-12)
  To compare carer burden between groups using the Caregiver Burden Scale (CBS)
2. Investigate potential moderators and mediators of effect at eight weeks, six months and twelve months
  To investigate whether baseline patient clinical characteristics and investigations (for example, routine brain CT scanning) help to predict those who might benefit from Co-careldopa augmented rehabilitation
  To investigate whether key factors (for example, fatigue measured by the Fatigue Assessment Scale (FAS)), concurrent musculoskeletal symptoms, signs and pain (using the MSK SSP manikin) and cognitive function (using the Montreal Cognitive Assessment (MoCA)) influence the short and long term effect of Co-careldopa on physical functioning
3. Investigation of implementation within NHS
  To assess the adverse event profile associated with Co-careldopa in addition to conventional NHS stroke rehabilitation treatment
  To investigate the practical implications of delivering this combined intervention within routine NHS acute and early community care of people with stroke
  To assess the acceptability of Co-careldopa treatment to stroke patients (study drug adherence will be measured)
4. To determine the cost-effectiveness of Co-careldopa versus placebo for rehabilitation after stroke

### Trial design

DARS is a multi-centre, prospective, randomised, double-blinded, placebo controlled trial of NHS physical therapy and occupational therapy treatment alone compared to NHS physical therapy and occupational therapy treatment with six weeks of Co-careldopa treatment for those admitted to acute stroke services after new or recurrent stroke.

A total of 572 people with stroke admitted to acute stroke services will be recruited. Each participant will be randomised to receive either Co-careldopa or placebo for six weeks post stroke. Routine NHS therapy will continue according to normal practice.

Outcome measures will be obtained at eight weeks, six months and twelve months following randomisation. The blinded design in which participants, treating clinicians and trial personnel will be blinded to group allocation will minimise bias by ensuring that Co-careldopa related intervention effects and information collection is the same between the active drug and placebo groups. Further minimisation of bias and maximising masking will be ensured by appropriate placebo and Co-careldopa preparation, blinding both patients and clinicians. Outcomes will be collected by assessors masked to the treatment allocation. All analyses will be undertaken blinded to treatment allocation until final analysis.

The National Research Ethics Service and the Medicines and Healthcare Products Regulatory Agency have approved this trial. Trial participants will be recruited from acute and community stroke services across more than 50 centres. Written informed consent will be obtained from all participants.

#### Setting

The trial will take place in stroke services with access to continuity of rehabilitation treatment following discharge from hospital. This can be through Early Supportive Discharge or hospital/community therapy according to local practice.

#### Inclusion criteria

1. New or recurrent clinically diagnosed ischaemic or haemorrhagic (excluding subarachnoid haemorrhage) stroke within 5 to 42 days prior to randomisation.
2. Cannot walk 10 metres or more indoors independently (that is, without use of physical assistance)
3. Rivermead Mobility Index score of <7.
4. Expected to need rehabilitation treatment
5. Aged 18 years or above
6. Able to give informed consent
7. Able to access continuity of rehabilitation treatment following discharge from hospital which is available within five days following hospital discharge.
8. Expected to be able to comply with the treatment schedule (for example, swallow whole tablets)
9. Expected to be in hospital for at least the first two doses of trial medication

#### Exclusion criteria

Patients meeting any of the following criteria are not eligible for trial entry.

1. Not expected to survive for two months following stroke
2. Diagnosis of Parkinson’s disease, severe medical or surgical illness, severe psychosis
3. Known hypersensitivity or contraindications to Co-careldopa
4. Symptomatic orthostatic hypotension
5. Needed physical assistance of at least one person to walk prior to stroke due to pre-existing co-morbidities (for example, heart failure, osteoarthritis)
6. Pregnancy, lactation or women of child-bearing potential unwilling to use medically approved contraception whilst receiving treatment and for one month after treatment has finished
7. Could not walk 10 metres or more indoors prior to their stroke (may have used a walking aid if necessary, but required no physical assistance). In this context physical assistance means help from one or more persons.

#### Recruitment and randomisation

**Figure 1:**
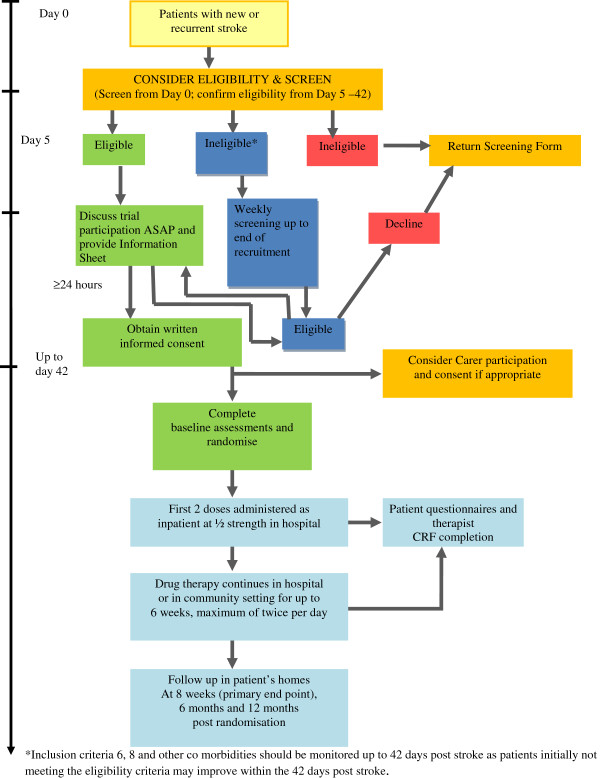
**Patient recruitment pathway.**

Patients admitted to stroke services after a new or recurrent stroke will be identified on admission to the stroke unit and considered for enrolment from day 5 to day 42 post stroke. Patients with aphasia will be given information in a manner that can be easily understood and, where practical, arrangements are made to meet the patient’s language, communication and other support needs. Patients who are initially ineligible for the trial due to other co-morbidities (for example, swallowing difficulties, lack of capacity to give informed consent) may subsequently become eligible. They will continue to be monitored until 42 days post stroke to determine if their condition improves and they consequently become eligible for the trial (refer to Figure 1: Patient Recruitment Pathway).

Informed written consent will be obtained from all participants and will also be sought from carers (if present) to allow information relating to carer burden to be collected.

Participants who fulfil the eligibility criteria will be randomised on a 1:1 basis to receive either Co-careldopa or placebo. Stratified randomisation will be used to ensure that treatment groups are well-balanced for: (1) recruiting centre; (2) type of stroke (primary intracranial haemorrhage; infarct); and (3) RMI score 0–3; >3 but <7.

#### Blinding

Patients, clinicians, site research staff and trial personnel at the Clinical Trials Research Unit (CTRU) involved in the day-to-day running of the trial will be blinded to group allocation until the final database lock. A matched placebo has been manufactured to match the commercial Co-careldopa. Final assembly, packaging and labeling of the Co-careldopa/placebo kit is performed by an independent clinical supplies company. Each kit is labeled and identified by a unique random five digit number only. A trial safety team will have access to the treatment allocation for the purposes of emergency unblinding and preparation of unblinded reports to the Data Monitoring and Ethics Committee.

#### Intervention

Patients will take a single oral tablet 45 to 60 minutes before routine NHS physical or occupational therapy sessions for a maximum of six weeks. Routine physical or occupational therapy is defined as active physical treatment (that is, most physical and occupational therapy directed at motor skills, such as walking, transfers, and dressing but not solely psychological input sessions or speech and language therapy). This also includes programmed rehabilitation delivered by rehabilitation assistants.

The dose and timing of the medication reflects current evidence on use of Co-careldopa in this context (9, 11, 12, 13). As part of this trial a pragmatic approach will be taken. Although the dose should be taken optimally between 45 to 60 minutes prior to the rehabilitation treatment session it is recognised that there may be occasions where, for example, the therapist is unable to contact the patient to remind them to take the tablet or the patient may forget. It is acceptable for the tablet to be taken within 0 to 15 minutes before the start of therapy in these situations. The peak effect of Co-careldopa is 0.5 to 2 hours after an oral dose. If the patient is scheduled to have two therapy sessions directly one after the other or within three hours of a dose then a repeat dose is not given before the second of the therapy sessions. If the patient is having more than two physical or occupational therapy sessions, the dose will not be administered more than twice during any one 24 hour period.

**Figure 2:**
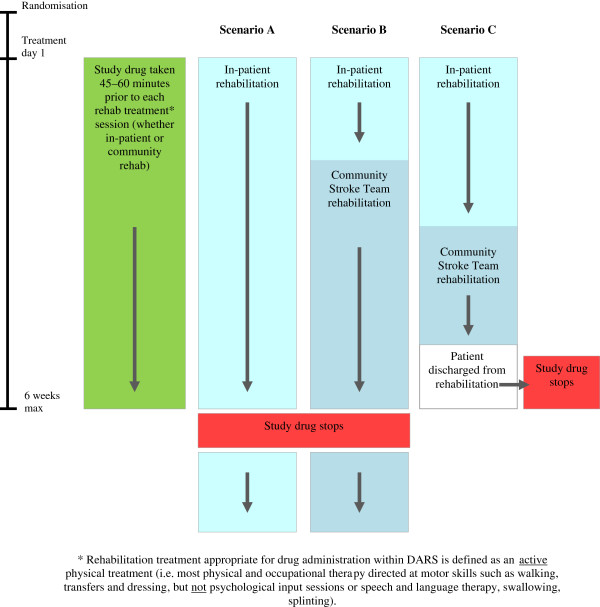
**Patient treatment scenario.**

Assuming a maximum of two sessions of physiotherapy (PT) or occupational therapy (OT) per day for 30 days over a six week treatment period, each patient will receive a maximum of 60 tablets during their participation in the intervention phase of the trial. Investigational Medicinal Product (IMP) with usual NHS rehabilitation treatments will be continued for a maximum of six weeks as long as it is deemed that the patient would benefit from ongoing rehabilitation. The duration of treatment may be less if the patient is clinically deemed not to require further rehabilitation treatment. The decision about need for rehabilitation interventions (when to start, finish and type) will be made by the treating clinicians, therapists and nurses in consultation with patients and families as part of the routine management of the patient (refer to Figure 2: Patient treatment scenarios). In both treatment arms, clinicians and therapists receive the same instructions with regards to need for interventions. The trial is blinded, ensuring both treatment groups receive the same amount of attention from clinicians and therapists. As part of the analysis we also assess the level of blinding by calculating blinding index for each treatment group.

The IMP will be first administered in an inpatient stroke unit after the patient has consented and been randomised. The first two doses of Co-careldopa will be 62.5 mg (Levodopa 50 mg and carbidopa 12.5 mg) and the remaining doses will be 125 mg (Levodopa 100 mg and carbidopa 25 mg) and will be administered in hospital. The participating site will, therefore, be able to closely observe patients for any early adverse events occurring as a result of the trial IMP. The patient will be assessed by the stroke unit nursing staff (prior to discharge from hospital) as to his/her ability to self-medicate (in relation to the IMP) after discharge from hospital. Once discharged from hospital (if prior to completion of the six-week course of IMP) the trial intervention will be continued at home or other discharge destination. Telephone reminders approximately one hour prior to a therapy visit will be provided by treating community rehabilitation staff to prompt the patient to take the trial medication prior to the community rehabilitation session. At the start of the therapy session, the rehabilitation staff will ask the patient if they have taken the IMP and what time the IMP was taken. The timing and content of the PT/OT session is recorded by the treating therapist. Where IMP has not been taken, it is recommended that the dose is taken as soon as the therapist arrives.

#### Outcomes

The primary outcome will assess whether patients can walk independently at eight weeks post-randomisation as measured by a RMI score of 7 or higher and can walk 10 metres with an aid if necessary but with no standby help (that is, answer ‘yes’ to item number 7 on the RMI).

Secondary patient outcomes (all measured at eight weeks, six and twelve months):

- *Physical functioning:* RMI (continuous), Barthel Index (dependency measure), Modified Rankin Scale, NEADL scale (extended activities of daily living), ABILHAND (bilateral arm activities)
- *Mood:* GHQ-12
- *Potential moderators and mediators of treatment effect*: baseline clinical characteristics and investigations (for example, routine brain neuroimaging) will be collected to predict those who might benefit from Co-careldopa augmented rehabilitation; fatigue (FAS)), concurrent musculoskeletal symptoms, signs and pain (using the MSK SSP manikin) and cognitive function (using the MoCA).
- *Implementation within NHS*: to investigate the practical implications of delivering this intervention within routine NHS acute and early community care of people with stroke; and to assess acceptability of Co-careldopa treatment to stroke patients through measuring study drug adherence will be undertaken with participants at the week 8 assessment.

Process outcomes: Standardised therapy forms will be used to document the type of rehabilitation received by the trial participant. These logs will also capture information ease of compliance with timing of treatment schedule, IMP packaging, clarity of instructions and labelling.

Carer outcomes*:* The CBS will be administered at eight weeks, six and twelve months to assess carer outcomes.

Safety: Adverse events, whether volunteered by the patient or carer, or identified by the therapist or researcher, until the eight week follow up appointment are documented. As an inpatient, the local research team will monitor the patient for the occurrence of adverse events. Following discharge from hospital, detection of most adverse events will occur through spontaneous reporting by patients, their carers, attending therapists or via the scheduled visits by the researcher. Serious adverse events (SAEs) will be reported to the CTRU up to 30 days after the last dose of protocol treatment. All serious unexpected suspected adverse reactions (SUSARs) are reported to the CTRU within 24 hours and expedited reported to the MHRA, ethics committee and Sponsor where applicable.

Health economics: Patient NHS resource use data (contact with primary, community and social care services together with hospital admissions and out-patient visits) will be collected along with EQ-5D health state utilities at baseline, eight weeks and at six and twelve months. Carers will also complete a resource use measure and the EQ-5D to respectively capture the costs and quality of life impact of providing care.

Assessments:

Patient outcomes at all follow-up time points (eight weeks, six and twelve months) will be collected via interview by the independent researcher in the patient’s home or at the hospital or community facility (refer to Table 1 Schedule of Events).

**Table 1:** **Schedule of events**

Assessment		Timeline (months post randomisation)		
	Baseline	8 weeks (+/- 7 days)	6 months (+/- 14 days)	12 months (+/- 14 days)
**Eligibility and consent**	X			
**Baseline data** (researcher/nurse completed from routinely collected data and ward staff)				
Rivermead Mobility Index (professional perspective on patient’s ability for stratification)	X			
Past medical history	X			
Lesion location and type (CT scan)	X			
Montreal Cognitive Assessment (MoCA)	X			
Randomisation (within 42 days post stroke)	X			
**Patient questionnaires** (completed via researcher interview with patient)				
Rivermead Mobility Index (patient’s perspective on ability)	X	X	X	X
ABILHAND scale	X^1^	X	X	X
Nottingham Extended Activities of Daily Living Scale	X^1^	X	X	X
General Health Questionnaire 12	X	X	X	X
EQ-5D	X	X	X	X
Barthel Index (postal version but collected face to face)	X	X	X	X
MSK-SSP Manikin	X^1^	X	X	X
Fatigue Assessment Scale		X	X	X
Health Economics Resource Use Questionnaire	X	X	X	X
**Carer questionnaires** (Carer completed)				
Caregiver Burden Scale		X	X	X
EQ-5D	X	X	X	X
Health Economics Resource Use Questionnaire	X^1^	X	X	X
**Qualitative follow-up**				
Patient/therapist perspective regarding use of IMP		X		
**Clinical follow-up data** (researcher/therapist/nurse completed)				
Treatment data (rehabilitation and drug compliance)		X		
Modified Rankin Scale		X	X	
Montreal Cognitive Assessment (MOCA)		X	X	X
Serious and non-serious adverse event monitoring	Continuous reporting as occur			
New significant medical/surgical illness (e.g. for stroke, myocardial infarction, cancer, fracture, elective surgical procedures)			X	X

^1^Pre stroke.

#### Sample size

We plan to recruit 572 patients from more than 50 hospitals across the UK. This will provide 90% power at 5% significance to detect 50% relative difference between the placebo and active treatment group in the proportion walking independently at eight weeks post randomisation (as measured by the RMI score 7 or greater and who also answer ‘yes’ to item number 7). This assumes the same control rate of 26% as in the Scheidtmann study [9] and will ensure the minimum improvement that can be detected is 39% of patients on active treatment who are walking independently by eight weeks.

The primary intention to treat (ITT) analysis will include all randomised patients as it will assume that patients who die or are lost to follow-up are unable to walk independently.

This sample size also provides 80% power to detect a small to moderate effect size of 0.3 in key secondary outcomes (for example, ABILHAND - to measure functional upper limb activities; the NEADL scale measuring instrumental activities of daily living, such as outdoor mobility and household tasks). It is important that the study has sufficient power to detect real change in these secondary outcomes given that they are (1) important functional parameters in addition to walking and (2) are also likely to change if the treatment is effective. For all secondary analyses, loss to follow up has been estimated at 10% at eight weeks (of those surviving stroke at two weeks), rising to 20% by twelve months.

#### Analysis

Statistical analysis is the responsibility of the CTRU statistician and a final statistical analysis plan will be written before any analysis is undertaken. All analyses will be conducted on the ITT population defined as all participants randomised regardless of non-compliance with the intervention. An overall two-sided 5% significance level will be used for all endpoint comparisons.

Primary analysis of independent walking ability at eight weeks post-randomisation (defined by a score of 7 or above and who also answer ‘yes’ to item 7 on the RMI) will be undertaken using logistic regression, while adjusting for gender, type of stroke, centre and RMI at baseline. For the primary outcome ITT analysis, it will be assumed that patients who die or are lost to follow-up are categorised as ‘unable to walk independently’. A sensitivity analysis will be undertaken to test the robustness of conclusions to this assumption. RMI will be analysed as a continuous measure as part of secondary analyses.

Other outcome measures will be analysed for each time point by regression models appropriate to the data type. Such analyses will adjust for patient-level covariates included as strata within the randomisation process including gender, type of stroke, centre and RMI. Appropriate methods will be used to handle missing data.

Potential predictors (fatigue, depression, pain, medical and surgical events, cognitive function, activities of daily living, number of rehabilitation therapy sessions and number of tablets taken) of response to Co-careldopa will be explored using baseline measurements taken for primary and secondary outcomes. In addition, we plan to model the relationship between potential moderator and mediator variables and treatment effect.

A per-protocol analysis will be carried out to indicate whether results are sensitive to the exclusion of patients who violated the protocol.

The number of patients reporting a SAE (up to 30 days after the last dose of treatment) and details of all SAEs will be reported for each treatment group.

The Trial Statistician will be blinded to treatment group allocation throughout the trial until the database has been downloaded for final analysis. Only the Safety Statistician, Supervising Trial Statistician, back-up Safety Statistician and Safety Data Manager will have access to unblinded treatment group allocation prior to final analysis.

The primary within-trial economic analysis will be a cost-utility analysis yielding cost per incremental quality-adjusted life year (QALY). A secondary within-trial analysis will estimate the incremental cost per patient achieving independent walking (as determined by a score of ≥7 on the RMI) at eight weeks post-randomisation for Co-careldopa versus placebo. A decision-analytic model will be developed to estimate the lifetime costs and effects (QALYs) of the interventions and the resulting incremental cost-effectiveness ratio (ICER) referenced to the NHS willingness to pay per QALY gain threshold. Extensive deterministic and probabilistic sensitivity analyses will be conducted to estimate the level of uncertainty around the results. QALY values will be based on EQ-5D utility and the perspective of the analysis will be Health and Personal Social Care.

#### Data monitoring

Trial supervision includes a core Project Team, Trial Management Group (TMG), a Trial Steering Committee (TSC) and Data Monitoring and Ethics Committee (DMEC). Only the DMEC will have access to unblinded data prior to final analysis and, in strict confidence, review unblinded adverse event data on a quarterly basis.

Data will be monitored for quality and completeness by the CTRU. Missing data will be chased until it is received, confirmed as not available or the trial is at analysis.

#### Trial organisation and administration

The DARS trial is funded by the Efficacy and Mechanism Evaluation (EME) programme (Grant reference number 08/43/61). The trial is sponsored by the University of Leeds and was developed by the DARS trialists in collaboration with the support of the UK Stroke Research Network Rehabilitation Clinical Studies Group. The trial is adopted by the UK Stroke Research Network and is supported in the delivery by the Stroke Research Network staff. The trial is registered (ISRCTN99643613; EudraCT Number: 2009-017925-20). The trial will be conducted in accordance with the EU Clinical Trials Directive 2001/20/EC and will be conducted in accordance with the UK Medicines for Human Use (Clinical Trials) Amendment Regulations 2006, the principles of good clinical practice (GCP) in clinical trials, the NHS Research Governance Framework, and through adherence to CTRU standard operating procedures. Ethical approval has been obtained through the UK National Research Ethics Service (ref 10/H1005/6).

## Discussion

Stroke has a huge impact, leaving more than a third of survivors with lasting disability. Rehabilitation remains the cornerstone treatment within the NHS and the current consensus suggests early rehabilitation interventions are likely to be of greater benefit. Therefore, enhancing the effect of conventional physical rehabilitation with L-dopa is likely to have greater impact when linked to early stroke care through a stroke unit and through community rehabilitation services. Co-careldopa provides an exciting and important opportunity to manipulate the brain’s pharmacological environment at a time when physiological remodelling of the brain is occurring through conventional rehabilitation treatments. This not only has potential to enhance the effect of conventional therapies but also new rehabilitation interventions. Understanding the relationship between pharmacologically primed neuroplasticity and practice dependent neuroplasticity is of major scientific interest in understanding how the brain adapts to injury. A key aspect of this type of intervention is temporally linking it to conventional rehabilitation treatments for people with stroke. The dose and timing of the medication within the trial design reflects current evidence on use of L-dopa in this context (9, 11, 12, 13). This study is the largest randomised controlled trial investigating the use of Co-careldopa to improve functional outcomes in acute stroke. If conclusive evidence of benefit is identified from this large trial, it is very likely that given the low cost of L-dopa preparations it will be adopted world-wide in stroke rehabilitation.

The clinical trial presented some implementation challenges that we have addressed (for example, pharmacoadherance; configuration of UK stroke services to allow continuity of intervention; challenges to recruitment).

Pharmacoadherance: A key aspect of this study has been to ensure that the drug is taken by the participant at the correct time both in hospital and when the person is at home. Stroke survivors may have significant residual impairments, such as weakness, aphasia, visual disturbance, cognitive problems and mood disorders, which may affect their ability to comply with the medication/therapy schedule. Non-adherence or partial adherence of research participants can result in inconsistent study results.

There is no single effective approach and, therefore, a multi-modal approach incorporating best available advice from the Cochrane review [14] and guidance provided by the American Psychological Society [15] has been used. This approach includes provision of understandable information sheets, reminders (for example, manual telephone follow-up for those in the community) and involving carers where appropriate. The available evidence suggests that the greater the interaction with the patient, the more likely the person is to adhere to the medication. This study also involves reasonably accurate timing of the dose (that is, prior to rehabilitation treatment). Mis-timing of medication is a common and important problem [16]. During trial set-up processes, we involved 19 stroke survivors in small group discussions about different aspects of IMP labelling and packaging. Different examples of IMP packaging, developed using UK National Patient Safety Association guidance, were presented to the patient group and preferences/opinions were obtained through standardised questionnaires. We have used push-through blister packs as they protect the study drug integrity. The packs are appropriately labelled so that the study drug is taken in relation to the rehabilitation sessions whether in the community or in hospital. The package will serve as a visual aid, encouraging patients to take the trial medication at the prescribed times and giving the patient the ability to recognize whether or not they have taken the scheduled dose. Prescription information and educational materials will be part of the medication’s packaging which will also include an instructional area on the blister pack itself; the outer carton of the package contains space for dosing instructions, reminders and branding in large, readable fonts. The nature of the optimal packaging and ensuring maximum pharmacoadherance was designed in collaboration with input from clinicians, pharmacy staff, people with stroke and their families and the manufacturer of IMP. This also includes design and implementation of appropriate training for those dispensing the trial intervention both in the hospital and when patients are self-medicating at home.

Monitoring adherence is a key process issue. Therapy staff, in conjunction with nursing staff if the participant is an in-patient, are asked to complete an intervention record for each participant which includes timing, therapy duration and type and whether the trial medication has been taken at the correct time. The research nurse at each recruiting centre will collate adherence information and complete Case Report Forms (CRFs) and send them to the CTRU. We will also undertake pill counting; even though it has limitations, it is suggested as a standard for monitoring medication adherence in clinical trials [17].

We have also developed a DVD for the participants/carers to view in the hospital or their home environment to provide an audio visual aid to supplement the Patient Information Sheet. The DVD includes voice over explaining trial processes, in particular, the therapy/IMP schedule, safety issues and contact details. IMP/therapy schedule compliance was also discussed with community therapists. The patient feedback was incorporated into IMP packaging to allow one handed opening and prompts for adherence to IMP schedule. The DVD content is presented in a manner accessible to patients with aphasia or hemi-sensory neglect and uses graphics to illustrate abstract concepts, such as randomisation. The DVD will be given to trial participants as part of the recruitment information pack.

A process has been implemented for the therapist (1) to call the patient 45 minutes before the therapy session to remind the patient to take their IMP and (2) to conduct a compliance check at each therapy session. Whilst an inpatient, the rehabilitation staff will liaise with nursing staff to ensure that patients are administered the trial medication prior to the rehabilitation treatments. Packaging is also a determinant in improving compliance rates. In the CRF for the study we have incorporated questions about these aspects to investigate participant opinion in the context of a clinical trial.

Configuration of UK stroke services: The English National Stroke Strategy is intended to provide a quality framework against which local services can secure improvements to stroke services and address health inequalities relating to stroke over the next ten years; provide advice, guidance and support for commissioners, strategic health authorities, the voluntary sector and social care in the planning, development and monitoring of services; and inform the expectations of those affected by stroke and their families by providing a guide to high-quality health and social care services. An important aspect of this strategy is the provision of early supported discharge from an acute hospital-based stroke service for people with moderate disability as a result of stroke. The exact configuration of the services is not crucial as long as the services are multidisciplinary and staff has the right specialist skills and can provide intervention without delay after discharge from hospital. A number of stroke services across England have implemented this aspect of the strategy. The configuration of these services can vary between those services that have a dedicated stroke-specific multidisciplinary team who provide ongoing rehabilitation in the person’s home to community hospital facilities which provide ongoing rehabilitation within an inpatient setting.

A key aspect of the design of the DARS trial is the recognition of the varied configuration of community stroke rehabilitation services as well as a system for continuity of provision of trial intervention after discharge from hospital given that the length of stay in hospitals for people with acute stroke is decreasing. The median length of stay of people with acute stroke in hospital in 2010 was 9 days and mean was 19.5 days [18]. The design of this clinical trial ensures patients are able to continue the trial medication after discharge from hospital to community. In the UK, community services are separate organisations to those of acute hospitals which can complicate the implementation of clinical trials of investigational medicinal products (CTIMPs) from a research governance perspective.

## Trial status

We anticipated to reach the target sample size by April 2014. Follow-up will continue for 12 months and we expect to publish the results in late 2015. The trial will be published in the NIHR Journals Library, which is open access, free to view and indexed on several databases including MEDLINE and the Cochrane Library. The dissemination strategy includes presentation of the primary results at relevant international stroke conferences, peer-reviewed publications and presentations at relevant stroke rehabilitation/neuroimaging conferences. We will work with the Public and Patient Involvement (PPI) representatives to develop lay reports to disseminate research findings to patient groups.

## Abbreviations

ABILHAND: ABILHAND manual ability measure
BI: Barthel index
CBS: Caregiver burden scale
CRF: Case report form
CTRU: Clinical trials research unit
DMEC: Data monitoring and ethics committee
ESD: Early supportive discharge
EQ5D: European quality of life-5 dimensions
FSS: Fatigue severity scale
GHQ-12: General health questionnaire 12 (12 item version)
ICER: Incremental cost-effectiveness ratio
IMP: Investigational medicinal product
ITT: Intention to treat
MoCA: Montreal cognitive assessment
MRS: Modified rankin scale
MSK-SSP: Manikin musculoskeletal symptoms/signs and pain Manikin
NEADL: Nottingham extended activities of daily living
NIHR EME: National institute of health research efficacy and mechanism evaluation
OT: Occupational therapy
PT: Physical therapy
QALY: Quality-adjusted life-year
RCT: Randomised controlled trial
RMI: Rivermead mobility index
SAE: Serious adverse event
SRN: Stroke research network
SUSAR: Suspected unexpected serious adverse reaction
TMG: Trial management group
TSC: Trial steering committee.
